# A case study to investigate the impact of overcrowding indices in emergency departments

**DOI:** 10.1186/s12873-022-00703-8

**Published:** 2022-08-09

**Authors:** Giovanni Improta, Massimo Majolo, Eliana Raiola, Giuseppe Russo, Giuseppe Longo, Maria Triassi

**Affiliations:** 1 Department of Public Health, University of Naples “Federico II”, Via Pansini, No. 5 - ZIP, 80131 Naples, Italy; 2 Interdepartmental Center for Research in Healthcare Management and Innovation in Healthcare (CIRMIS), University of Naples “Federico II”, Naples, Italy; 3 A.O.R.N. “Antonio Cardarelli”, Naples, Italy

**Keywords:** Overcrowding in emergency department (ED), ED evaluation indices, National Emergency Department Overcrowding Scale (NEDOCS), Emergency department work index (EDWIN)

## Abstract

**Background:**

Emergency department (ED) overcrowding is widespread in hospitals in many countries, causing severe consequences to patient outcomes, staff work and the system, with an overall increase in costs. Therefore, health managers are constantly looking for new preventive and corrective measures to counter this phenomenon. To do this, however, it is necessary to be able to characterize the problem objectively. For this reason, various indices are used in the literature to assess ED crowding. In this work, we explore the use of two of the most widespread crowding indices in an ED of an Italian national hospital, investigate their relationships and discuss their effectiveness.

**Methods:**

In this study, two of the most widely used indices in the literature, the National Emergency Department Overcrowding Scale (NEDOCS) and the Emergency Department Working Index (EDWIN), were analysed to characterize overcrowding in the ED of A.O.R.N. “A. Cardarelli” of Naples, which included 1678 clinical cases. The measurement was taken every 15 minutes for a period of 7 days.

**Results:**

The results showed consistency in the use of EDWIN and NEDOCS indices as measures of overcrowding, especially in severe overcrowding conditions. Indeed, in the examined case study, both EDWIN and NEDOCS showed very low rates of occurrence of severe overcrowding (2–3%). In contrast, regarding differences in the estimation of busy to overcrowded ED rates, the EDWIN index proved to be less sensitive in distinguishing these variations in the occupancy of the ED. Furthermore, within the target week considered in the study, the results show that, according to both EDWIN and NEDOCS, higher overcrowding rates occurred during the middle week rather than during the weekend. Finally, a low degree of correlation between the two indices was found.

**Conclusions:**

The effectiveness of both EDWIN and NEDOCS in measuring ED crowding and overcrowding was investigated, and the main differences and relationships in the use of the indices are highlighted. While both indices are useful ED performance metrics, they are not always interchangeable, and their combined use could provide more details in understanding ED dynamics and possibly predicting future critical conditions, thus enhancing ED management.

## Introduction

The emergency department (ED) is an important public service that provides immediate access and stabilization for patients with emergency conditions [1]. The immediate access nature of the ED has led an increasing number of people to use it as a preferential route to access treatment, causing overcrowding. ED overcrowding, more correctly defined as access block, has become a worldwide phenomenon, causing serious consequences on patient outcomes, staff work and the system, with a general increase in costs [2].

The problem of crowding (or overcrowding) of the ED, as well as that of long patient wait times, occurs domestically and internationally. Several techniques have been adopted to improve the efficiency of the procedures in the ED [3, 4]. In recent years, in particular, there has been an increasing use of data analysis and artificial intelligence to enhance biomedical data and signal analysis, for example as support for diagnosis [5, 6], for the development of simulation models to support the characterization of flows [7–9] or for the optimization of processes through the support of appropriate performance indicators [10, 11]. There are several studies that have provided for the implementation of these techniques directly in the ED, for the study of waiting times [12], length of stay [13, 14] and drop-out rates [15, 16]. The ED, with its complex mission, namely, that of providing an adequate, timely and optimal response to patients who present themselves in an unscheduled manner and addressing clinically critical situations by implementing all the necessary life-saving practices [17], needs even more rigorous analysis and efficiency. The main problem is that there is no universal standard definition of overcrowding in emergency rooms because there is no single standard measure of hospital performance [18, 19]. One of the definitions that seems more complete is that provided by the American College of Emergency Physicians (ACEP) Crowding Resources Task Force, according to which overcrowding can be defined “*a state in which the identified need for emergency services exceeds available resources in the ED. This situation occurs in hospital EDs when there are more patients than staffed ED treatment beds and when wait times exceed a reasonable period*” [20].

ED overcrowding is often measured through the mean occupancy rate or by dividing the number of patients in the emergency department by the number of treatment spaces [21].

In recent years, a large number of scientific studies have addressed this problem, including contributions from different areas of research [22, 23]. In 1990, the United Kingdom became the first country to introduce a few clinical indices [24] based on the quickness in assessing the condition of the patient as a parameter [25]. However, in 2010, Jones and Schimanski [26] demonstrated that the introduction of an ED time target effectively abolished this measure [27], and the associated massive financial investment has not resulted in a consistent improvement in the United Kingdom. Therefore, new indices have been proposed [24] to indicate the quality of care through a wider range of variables that can be monitored: 1) time to initial nursing review, 2) duration of treatment, 3) number of outpatients, and 4) number of patients who leave the department without being seen. A problematic point, however, was that the theoretical indices usually did not reflect the actual conditions in which clinicians found themselves working. Zhou et al. [28] collected subjective and objective emergency department occupancy (EDO) data three times a day (1:00, 9:00, and 17:00) over a period of six months and analysed them using Bland–Altman and Kappa tests. The results showed that the NEDOCS (National Emergency Department Overcrowding Scale) index did not consistently reflect the sense of overcrowding perceived by the staff in the emergency room, calculated by the VAS (Visual Analogue Scale) method. This situation highlights the importance of identifying a complete taxonomy of the ED crowding indices present in the scientific literature.

The scientific literature identifies 16 main indices of ED crowding, including four multidimensional indices (EDWIN [Emergency Department Work Index], Hazard Stairs, READI [Real-time Emergency Analysis of Demand Indicators], and NEDOCS), five input indices (total capacity for first aid, number of patient arrivals in six hours, ambulance transport number, number of patients waiting for medical treatment, and number of patients in the waiting room), three indices of throughput (length of stay in the emergency room [ED LOS], wait time for a first appointment, and time spent in the waiting room), and two indices of output (number of patients in the emergency room and percentage of total beds occupied) [29].

Once the indices were defined, various studies were conducted to validate them. Tekwani et al. [30] conducted a survey based on an interval of eight months on a sample of patients discharged from the emergency room to quantitatively assess the effect of crowding on patient satisfaction using a variety of questions associated with a Likert scale score, a widely used scale for healthcare quality assessment studies [31, 32].

McCarthy et al. [33] compared the emergency room occupancy rate, calculated as the ratio between the total number of hospital stays and the total number of hospital beds in a given period, to measure emergency room crowding, with a validated EDWIN index. Although not extremely accurate, the latter index can be used to quantify crowding and has the advantage of being simpler and more intuitive than the other indices. Several studies were also conducted to assess the correlation between the EDWIN score and the frequency of medical errors [34] or the delayed antibiotics for sepsis [35]. In a study by Todisco [36], after the introduction of six beds into the emergency room, there was a 10.11% reduction in the NEDOCS. There are also several examples of the application of the NEDOCS index to measure ED crowding [19, 37, 38].

Several works have also compared the performance of the EDWIN and NEDOCS indices in evaluating overcrowding. Weiss et al. [39] demonstrated that both indices, and in particular the NEDOCS index, show good accuracy in measuring the overcrowding of an emergency room. Instead, Bernstein et al. [40] showed a strong correlation between the EDWIN index and the staff’s crowding assessment.

This work aims to correlate the EDWIN and NEDOCS indices to verify their validity for evaluating ED crowding at the “A. Cardarelli” Hospital of Naples. The possibility of including a run-time instrument in the information system of the ED is also considered.

## Methods

### Study design, setting, and population

This is a prospective study of the assessment mechanisms for ED overcrowding. This study did not involve any patient contact, and all identifiers were removed from the clinical information. This study was performed at the ED of the hospital “A. Cardarelli” of Naples, representing a centre of national relevance and the largest hospital in southern Italy. The mission of the National Importance Hospital “Antonio Cardarelli” is to be an acute care hospital that provides highly effective and quality diagnosis, treatment, and rehabilitation services; it offers emergency services (with approximately 300 daily accesses) and, in election, ordinary hospitalization, day surgery, day hospital and outpatient services. In 2021, the structure had 1060 beds in assets divided between the 99 different medical specialties present and related to eight health departments.

Our study included 1678 records that included all patients who presented to the ED over a seven-day period.

### Measurement of overcrowding

To evaluate an overcrowding measure of the ED of the hospital “A. Cardarelli” of Naples, the ED status was recorded every 15 minutes for a period of 7 days from March 14th to March 20th, 2016. Overcrowding conditions occurred when the number of patients at the ED exceeded the number of beds available. The NEDOCS and EDWIN indices were calculated using values that were available for download from the hospital’s system database, which easily presents the numbers of patients and admissions. The ED system is updated throughout the patient flow. Upon the patient’s arrival in the ED, information such as the patient’s personal details, medical history and reason for admission is collected. This initial part is completed with the assignment of a triage colour code (red, green, yellow or white depending on the severity of the medical condition) which is entered manually by the triage doctor after careful assessment. In addition, the ED patient file contains all information and data (results of diagnostic and laboratory tests, report of the medical examination, time from admission to first and second triage, first and second colour code assigned, time from admission to discharge/hospitalisation, type of admission).

The 15-minute period allowed the computerized system to acquire a snapshot of the ED status to determine the conditions of the different patients.

### Measurement of overcrowding through the EDWIN index

The EDWIN index is defined as [41]:

$$\frac{\sum_i{n}_i\ast {t}_i}{N_a\ast \left({B}_T-{B}_A\right)}$$

where

- n_i_: Number of patients in the emergency room in the i-th category of triage
- t_i_: Triage category (scale of 1 to 4, where 4 is the gravest)
- N_a_: Number of attending physicians on duty
- B_T_: Number of treatment beds
- B_A_: Number of patients in the ED

The number of patients in the emergency room is divided based on the corresponding category of triage. t_i_ is 1 for patients with the white code, 2 for patients with the green code, 3 for patients with the yellow code, and 4 for patients with the red code. The number of attending physicians on duty is 4.

The values of the EDWIN index are displayed considering the different values for each of the 7 days of the considered week. An active but manageable ED is represented by an EDWIN score less than 1.5, a busy ED is represented by an EDWIN score between 1.5 and 2, and a crowded ED is represented by an EDWIN score greater than 2.

### Measurement of overcrowding through the NEDOCS index

The NEDOCS index compiled by Weiss et al. [41] in 2004 is defined as

$$-20\ast 85.8\ast \left(\frac{TP}{ED\ Bds}\right)+600\ast \left(\frac{Brdg}{H\ Bds}\right)+13.4\ast (Vent)+0.93\ast \left( Long\ Admt\right)+5.64\ast (LBT)$$

where the variables are as follows:

- TP: Total number of patients present in the emergency room
- ED Bds: Total number of beds in the ED
- Brdg: Total number of patients waiting for treatment
- H Bds: Number of accredited hospital beds (Table 1)
- Vent: Number of patients undergoing respiratory care
- Long Admt: Longest wait time (in hours) for patients awaiting treatment
- LBT: Wait time of the last patient called from the waiting room (door-to-bed)

**Table 1:** Number of accredited beds at the hospital “A. Cardarelli” of Naples

DEPARTMENTS	Day Hospital BEDS	Hospitalization BEDS
CARDIOLOGY	2	24
GENERAL SURGERY	8	139
MAXILLO FACIAL SURGERY	2	14
PAEDIATRIC SURGERY	2	8
PLASTIC SURGERY	2	12
THORACIC SURGERY	1	10
VASCULAR SURGERY	2	14
HEMATOLOGY	24	35
GENERAL MEDICINE	11	165
NEPHROLOGY	1	7
NEUROSURGERY	2	58
NEUROLOGY	4	27
OPHTHALMOLOGY	4	14
ORTHOPEDICS AND TRAUMATOLOGY	7	77
OBSTETRICS AND GYNECOLOGY	7	40
ENT	2	16
PAEDIATRICS	10	32
TOXICOLOGY	0	4
UROLOGY	4	24
BURN UNIT	0	15
INTENSIVE CARE	2	30
CORONARY UNIT	0	18
CASUALTY DEPARTMENT	0	40
DERMATOLOGY	2	10
RECOVERY AND FUNCTIONAL REHABILITATION	2	13
GASTROENTEROLOGY	3	32
ONCOLOGY	10	21
PNEUMOLOGY	6	34
NEONATAL INTENSIVE CARE	2	17
**TOTAL**	**122**	**950**
**TOTAL**	**1072**	

NEDOCS index values from 0 to 50 indicate a normal situation in the ED, values from 50 to 101 indicate busy, values from 101 to 140 indicate overcrowded, values from 141 to 180 indicate severe overcrowding and values > 180 indicate disaster.

### Comparison between EDWIN and NEDOCS indices

Correlation coefficients were calculated to test the associations between the two overcrowding indices. First, normality was assessed by means of the Shapiro–Wilk test (95% confidence interval). Due to the nonnormal distribution of the data, Kendall’s correlation coefficient (τ) was used as a measure of correlation between the two indices. The reference ranges for Kendall’s correlation coefficient (τ). In accordance with [42, 43] we adopted the following ranges: |τ| < 0.10: very weak; 0.10 < |τ| < 0.40: weak; 0.40 < |τ| < 0.70: moderate; |τ| > 0.70: strong. Validation of the comparison between the two indices in terms of reliability and effectiveness was carried out with the support of ED staff, i.e., through a brainstorming session and periodic meetings to analyse the measurement obtained with the tested indices in the framework of the daily clinical practice and management of the ED. Along the observation period, there was a constant collaboration with the ED staff and the monitoring and analysis of the obtained results have been assessed on a per day basis. During the brainstorming sessions, the possible organizational issues in the ED workflow have been discussed with the ED staff. A meeting at the end of each day has been carried out to examine the outputs of the analysis through both EDWIN and NEDOCS indicators.

## Results

**Fig. 1:**
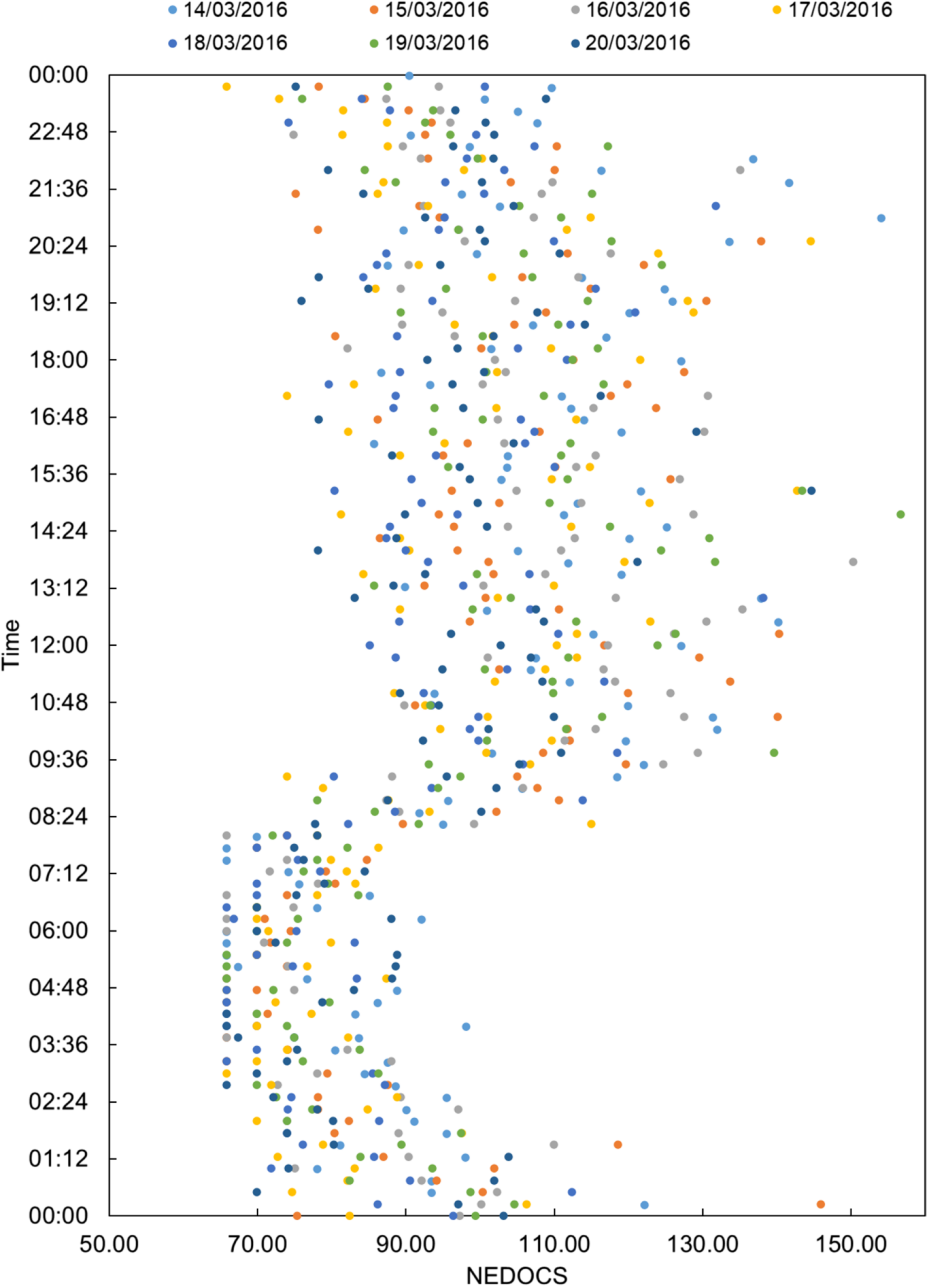
NEDOCS index values for the emergency department at the hospital “A. Cardarelli” hospital over a period of 7 days

The NEDOCS index results reported in Fig. 1 show the complete absence of the normality condition (0 < NEDOCS < 50). Over the entire week, the ED status results were similar among the seven days, and the majority of the time, the ED status was busy (51 < NEDOCS < 100). From 9:36 am to 00:00 am, the NEDOCS index values were in the range of 141–180, indicating severe overcrowding.

**Fig. 2:**
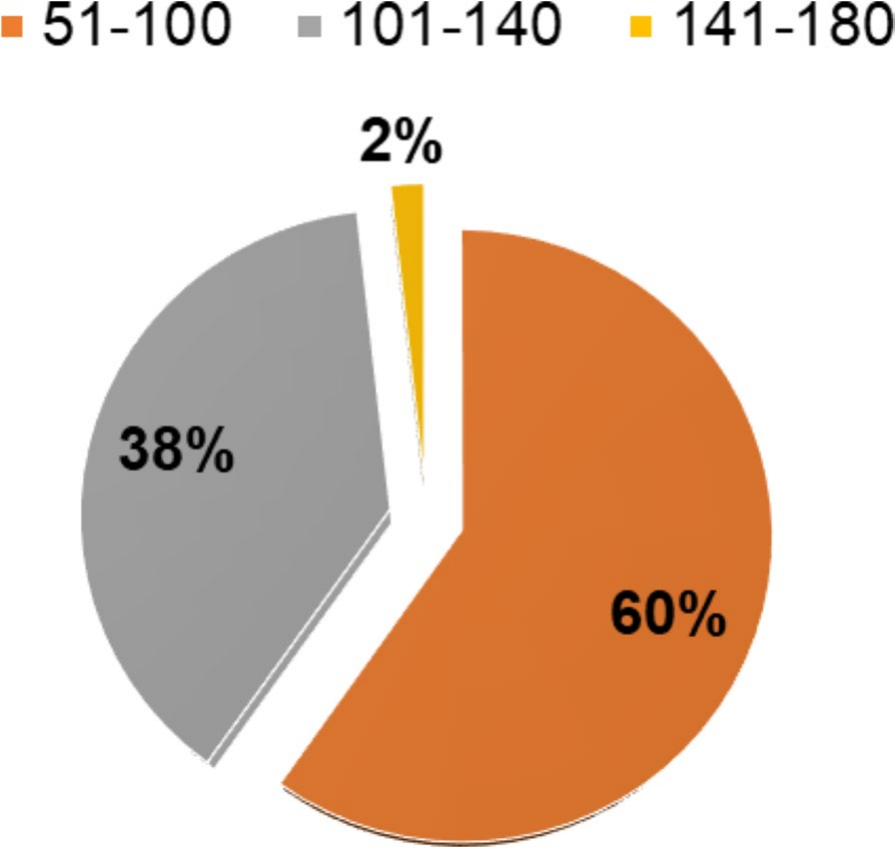
NEDOCS index values for March 14th, 2016

Considering a single day, March 14th, 2016, 0% of the NEDOCS index values were in the 0–50 range, 60% were in the 50–100 range, 38% were in the 100–140 range, and 2% were in the 140–180 range (Fig. 2).

**Fig. 3:**
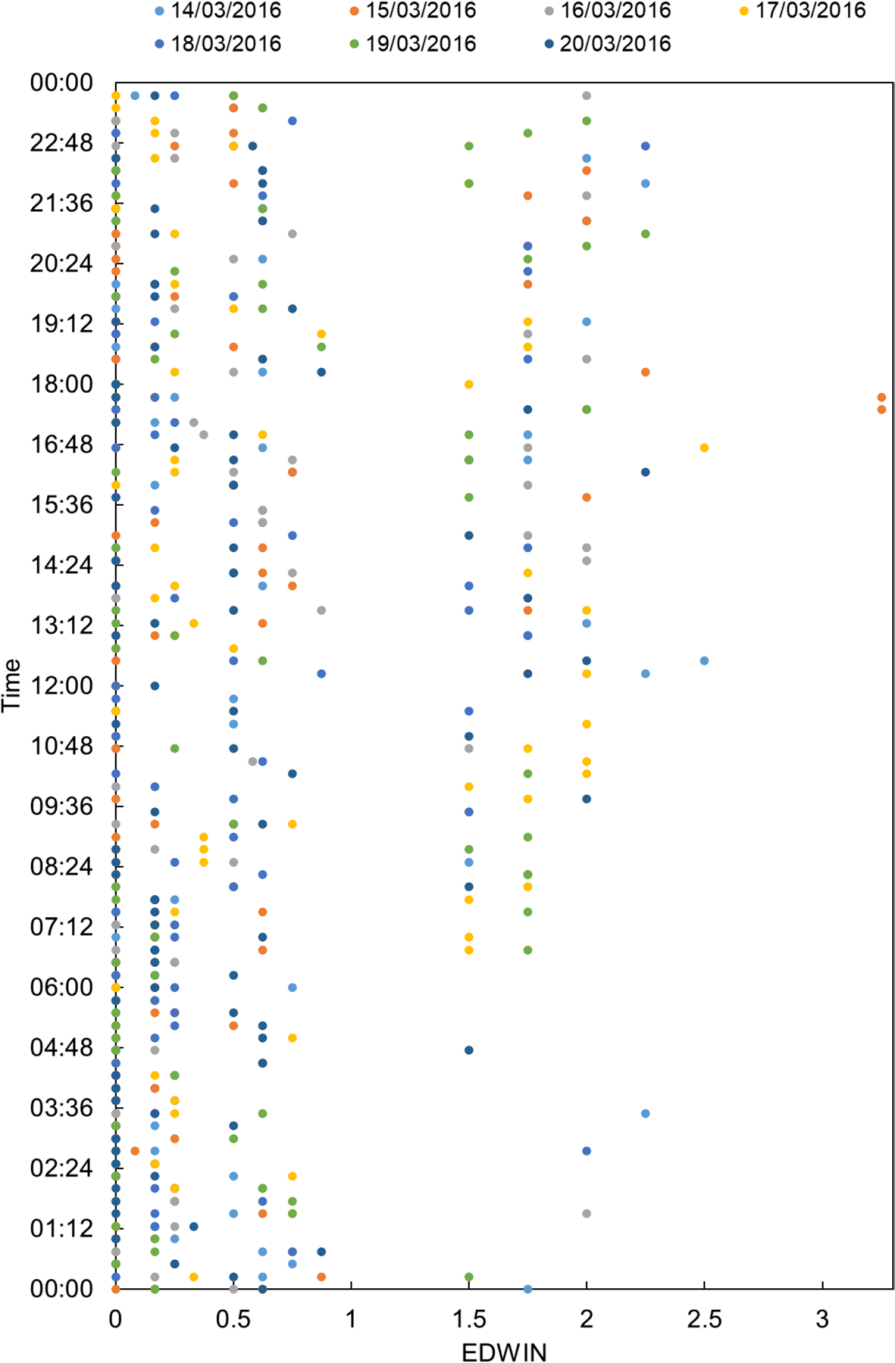
EDWIN index values for the emergency department at the hospital “A. Cardarelli” hospital over a period of 7 days

The EDWIN index reported in Fig. 3 was calculated for seven days, with the ED status recorded every 15 minutes.

For the EDWIN index calculations, when the BT (number of treatment bays) was lower than or equal to the BA (number of patients in the ED), we obtained a negative or zero value.

**Fig. 4:**
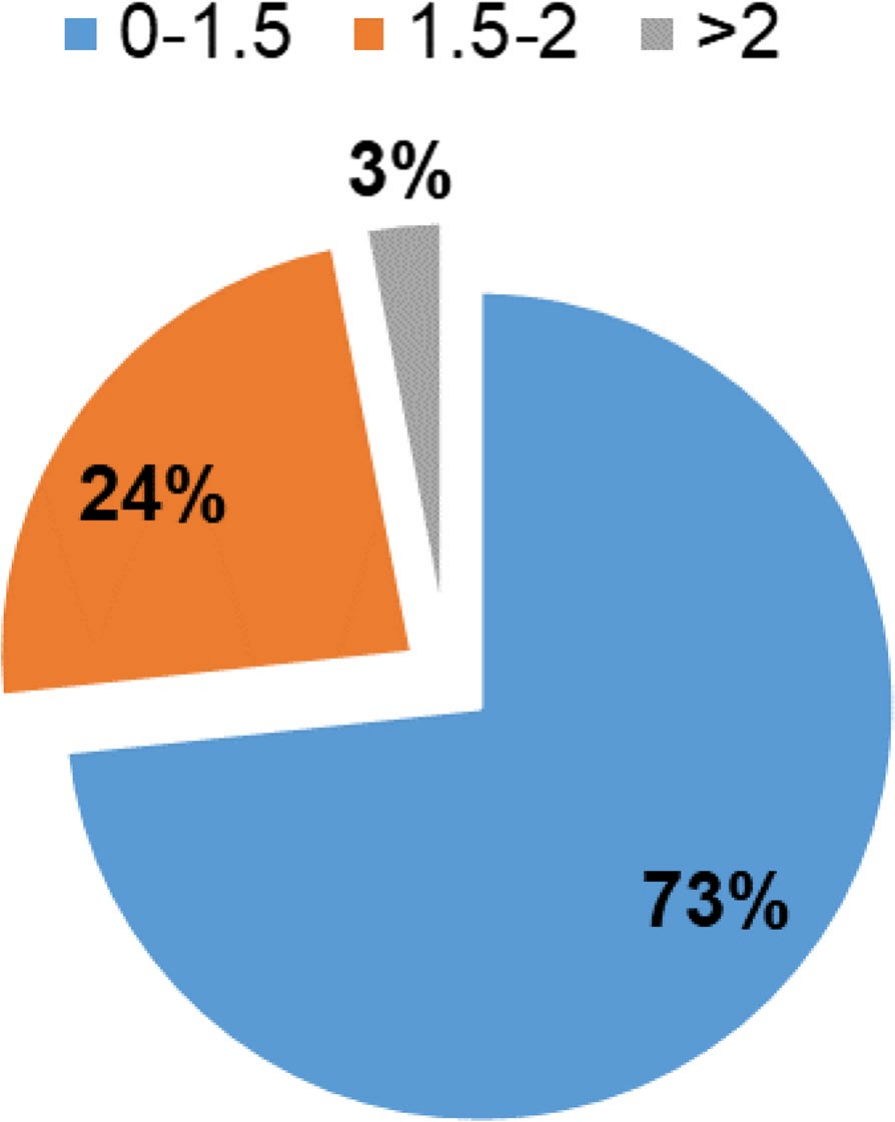
EDWIN index values for March 14th, 2016

As performed with the NEDOCS index, the EDWIN index was calculated for both seven days and a single day (Figs. 3 and 4, respectively).

Figure 3 shows a consistent situation over the seven days. During the weekend, the ED status was manageable, with values of < 1.5, whereas during the week, the EDWIN index values were in the 1.5–2 range from 7:00 am to 00:00. This pattern differed from that observed during the entire week at different time points. In particular, within certain time windows (e.g., from 9:30 to 00:00), more severe overcrowding occurred.

As performed with the NEDOCS index, we also considered a single day, i.e., March 14th, 2016 (Fig. 4). The results show that 73% of the EDWIN index values ranged from 0 to 1.5 and that 3% of them were > 2.

**Fig. 5:**
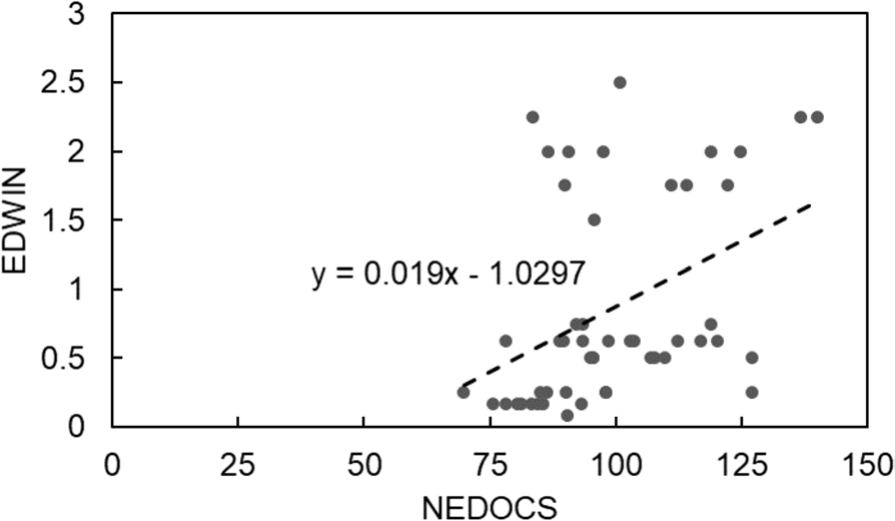
Comparison between the Emergency Department Work Index (EDWIN) results and the National Emergency Department Overcrowding Scale (NEDOCS) results for a selected day (March 14th, 2016). A linear regression curve (dashed line) is displayed to show the slight monotonic relationship between the two indices

Figure 5 shows a comparison between the EDWIN and NEDOCS indices on a single day, March 14th, 2016. Normality checks were carried out using the Shapiro–Wilk test before calculating the correlation coefficient. Due to the no normal distribution of the data (*p value* < 0.005), we verified conditions to apply Kendall’s correlation coefficient.

Therefore, after verifying that the data for both EDWIN and NEDOCS were measured on a continuous scale, the values of the indices were paired, which revealed a monotonic relationship between the data (as shown in the scatter plot in Fig. 5), and a Kendall’s coefficient of 0.36 was obtained, demonstrating a weak positive correlation between the two indices.

## Discussion

In this work, ED data relating to the activity of the hospital “A. Cardarelli” of Naples (Italy) were studied. Crowding was analysed at 15-minute intervals for a period of 7 days from March 14th to March 20th 2016 by calculating the EDWIN and NEDOCS indices. The problem of overcrowding, recognized worldwide, is generally caused by entities outside the ED. Lack of beds for admitted patients, lack of access to primary care and specialist physicians, shortage of ED nursing and physician staff, high complexity and triage acuity of patients are just some of the causes that contribute to the problem [44]. In our study indices were used, such as NEDOCS and EDWIN, which contain information such as the number of beds, triage category and number of treating doctors in service, which allowed us to increase our knowledge regarding this problem. In particular, it is highlighted that the ED is more crowded in the morning and in the midweek days. Being able to obtain an accurate forecast can be useful not only to improve the daily activity but also to know about critical situations in advance and to distribute resources accordingly in a better way. This could also be useful in identifying corrective actions to be taken in the most critical situations. Solutions could include an increase in inpatient beds or a greater number of resources in the hours in which a greater number of accesses is expected. For example, it has been shown that recording the patient in bed rather than in front of the ED reduces waiting times; however, at least in some places, this practice is not supported [45].

The results show that NEDOCS was slightly superior in measuring overcrowding. Hence, we demonstrate that the EDWIN and NEDOCS values are weakly correlated with one another through the measurement of Kendall’s correlation coefficient, which was equal to 0.36. In the examined case, a weak (0.36) is achieved. The obtained results show not only that both EDWIN and NEDOCS indices are consistent in the respective measurements of overcrowding, which can be seen as an indirect measure of typical ED variables (e.g., workload of emergency health care staff, delay in clinical decision-making, longer wait times, poor health care outcomes for patients, etc.) and that they are weakly correlated with one another but also that a slight superiority of the NEDOCS index could be hypothesized over the EDWIN index in measuring overcrowding in discriminating different crowding degrees thanks to its deeper and larger scale, which allows fine and more accurate measurements of the conditions at the interface between busy and crowded EDs (as in the comparison of Fig. 2 vs. Fig. 4). Indeed, while both indices show consistency in determining the very low rate of occurrence of severe overcrowding (2–3%), regarding differences in the estimation of busy to overcrowded ED rates, the EDWIN indices proved to be less sensitive in distinguishing these variations in the occupancy of the ED, classifying a busy ED in 73% of the cases, while from both brainstorming sessions with ED staff and from the NEDOCS measurements, a more reasonable percentage of busy ED condition is achieved with NEDOCS (60%).

Although the study of correlation between indices is a novelty in our work and offers the possibility of studying the joint effects of several variables, our work is not without limitations. A denser sampling offers our study strong innovation, but the short observation period limits its potential. Moreover, this preliminary evaluation relies also on the subjective opinion of ED staff involved in the study, which, in this case, was considered as the reference term for the assessment of the obtained results and the comparison of the two indices tested, since it represents the voice of experienced health professionals constantly working in the ED. A more robust verification procedure will be designed and implemented in further studies. In addition, no further external effects were considered, such as possible delays due to the occupation of the beds in the hospital wards. Finally, the study analysed data for 2016; therefore, current effects such as the COVID-19 pandemic are not included.

## Conclusions

In summary, the EDWIN and NEDOCS indices are used to analyse the activities of the ED of the national hospital “A. Cardarelli” of Naples (Italy). The results obtained from the implementation over a typical week confirmed the effectiveness of both indices in measuring overcrowding of the ED, highlighting time-based trends of crowding conditions and classifying them according to a predefined scale. It emerged that overcrowding occurred more often in the middle of the week, presumably causing a longer time to treatment. The two adopted indices were demonstrated to be weakly correlated, with NEDOCS being slightly more accurate in discriminating between busy and crowded ED conditions. Although it was not possible to identify the causes contributing to the problem, given the limited observation period considered, the achieved results confirm that the combined use of EDWIN and NEDOCS, despite not being fully interchangeable with one another, could provide more details in understanding the ED dynamics and possibly predict future critical conditions based on more accurate knowledge of the current and past trends in ED crowding, thus enhancing ED management.

## Data Availability

The datasets generated and/or analysed during the current study are not publicly available for privacy reasons but could be made available from the corresponding author upon reasonable request.

## Abbreviations

DH: Department of Health
EDs: Emergency Departments
NEDOCS: National Emergency Department Overcrowding Scale
EDWIN: Emergency Department Work Index
VAS: Visual Analogue Scale
EDO: Emergency Department Occupancy
READI: Real-time Emergency Analysis of Demand Indicators
ED LOS: Length of Stay in the Emergency Department
ESI: Emergency Severity Index
